# Role of Physiotherapy in Pediatric Lissencephaly: A Case Report and Therapeutic Insights

**DOI:** 10.7759/cureus.62901

**Published:** 2024-06-22

**Authors:** Anam R Sasun, H V Sharath

**Affiliations:** 1 Department of Neurophysiotherapy, Ravi Nair Physiotherapy College, Datta Meghe Institute of Higher Education and Research (Deemed to be University), Wardha, IND; 2 Department of Paediatric Physiotherapy, Ravi Nair Physiotherapy College, Datta Meghe Institute of Higher Education and Research (Deemed to be University), Wardha, IND

**Keywords:** pediatric rehabilitation, dysphagia, hippotherapy, neurodevelopmental techniques, lissencephaly

## Abstract

Type 1 lissencephaly is a genetic disorder of chromosomal abnormality. This case report glimpses at the physiotherapy rehabilitation for a two-year-old male brought by his parents with complaints of being unable to move his upper and lower limbs, delayed milestones as compared to his peer group, and difficulty in swallowing. Physiotherapy rehabilitation included Rood's approach to neurodevelopmental techniques, hippotherapy, vestibular ball rehabilitation exercises, oral sensorimotor stimulation, and tactile stimulation. The protocol lasted for 12 weeks. At the end of the rehabilitation, there was a significant improvement in the tone of the muscles and delayed developmental milestones. Through this case report, we conclude about the importance of genetic counseling to the parents of genetic disorders babies. We ought to improve awareness about the pivotal role of physiotherapy in managing such disorders. We conclude that physiotherapy significantly improved the symptoms and improved the quality of life of patients with type 1 lissencephaly.

## Introduction

Lissencephaly or “smooth brain” is a rare genetic developmental disorder caused by the arrest or defect of neuronal mitigation [1]. Both hereditary and non-genetic factors are the causes. Non-genetic variables include viral infections, primarily during the first trimester, that the mother contracts, and inadequate oxygenated blood supply to the brain during fetal development [2]. Disorders resulting from widespread irregular trans-mantle migration, such as subcortical band heterotopia, agyria, and pachygyria, together comprise the complex range of lissencephaly [3]. Excessively wide gyri, cortical thickness, disarray, and misplaced neurons in the subcortical white matter, including agyria, pachygyria, and gray matter heterotopia, are the main characteristics of lisencephaly [4]. The genetic factors known to cause lissencephaly are DCX (double cortin), gene TUBA1A (tubulin alpha 1a), ACTB (actin beta), ACTG1 (actin gamma 1), and ARX (aristaless-related homeobox). In recent years, the development of advanced molecular genomics technologies has led to the discovery of numerous additional genes [5].

Although the precise prevalence of lissencephaly is unknown, estimates put it anywhere from 11.7 and 40 per million [6]. There is an unknown prevalence of milder phenotypes of lissencephaly, with a prevalence of the classic type being 11.7 per million births (one of 85,470) [7]. The clinical features include developmental delay, epileptic seizures, hypertonia, facial dysmorphism, microcephaly, dysphagia, psychomotor retardation, spastic quadriplegia, and hypotonia with exaggerated tendon reflexes [8]. Additional characteristics include a tiny jaw, protruding upper lip, bi-temporal hollowing, short upturned nose, high forehead, and low weight for age. Children with lissencephaly undergo supportive care with the goal of symptom reduction.

During embryogenesis, the migration of post-mitotic neurons from the ventricular zone to the cortical plate stands as a pivotal stage in brain development. When this process is deficient, it frequently leads to significant brain malformations, such as lissencephaly [9]. Despite thorough diagnostic evaluations, a considerable number of individuals with malformations of cortical development continue to lack a molecular diagnosis. Addressing the intricate nature and significant clinical and genetic diversity of these malformations requires specialized and multidisciplinary expertise [10]. Prenatal diagnosis can be achieved by identifying abnormal development of sulci and gyri, with magnetic resonance imaging (MRI) serving as a valuable tool in detecting developmental cortical disorders and ocular anomalies [11]. The procedure of referring children with genetic diseases to physical therapy is sometimes unclear to carers. To encourage adherence to physiotherapy sessions and rehabilitation programs, it is imperative to provide carers with information regarding the benefits of physical therapy for children with genetic abnormalities [12]. In this study, we aim to report a case of a two-year-old male child diagnosed with type 1 lissencephaly and the role of physiotherapy in managing the complaints.

## Case presentation

A two-year-old male child was brought to the pediatric physiotherapy outpatient department with concerns from his parents regarding parental delayed developmental milestones, increased tone in bilateral upper and lower limbs, and difficulty in swallowing over the past year. The child was born through natural vaginal delivery at 40 weeks and three days of pregnancy and weighed 2.70 kilograms. His Apgar (appearance, pulse, grimance, activity, and respiration) scores at one minute and five minutes were 6 and 8, respectively. The baby did not cry immediately after birth and required resuscitation with tactile stimulation and nasal prong oxygen for four days. At six months of age, the baby started developing focal seizures occurring three to six times per week. He was treated conservatively with phenytoin. The patient exhibited an increasing number of tonic movements and ocular flashing episodes, which were followed by postictal behavior. The patient increasingly showed episodes with the flickering of eyes and tonic movements followed by postictal behavior. Following the onset of seizures, the baby exhibited regression in developmental milestones, decreased head control, rolling, and sitting without support and regression in grasping objects, reaching for objects, and transferring objects from one hand to another. Further investigations including MRI, electroencephalography, and a complete blood profile were conducted, which revealed a diagnosis of type 1 lissencephaly based on the MRI report.

Examination

After obtaining oral consent from the mother, a physical examination was done. The patient was vitally stable at the time of examination. Respiratory system examination did not reveal any obvious abnormalities. The physiotherapy examination is demonstrated in (Table 1).

**Table 1:** Examination of the patient cm: centimeter; kg: kilogram

Sr. no.	Examination	Findings
1	Anthropometric measurement:	
	Length	78 cm
	Weight	9.4 kg (ectomorphic)
	Head circumference	39 cm
	Chest circumference	40 cm
2	Higher cortical functions:	
	Overall activity	Average
3	Developmental milestones:	
	Gross motor	Presence of partial head control
	Fine motor	Presence of grasp and release (immature)
		Presence of pincer grip (immature)
	Language	Turns head to sound and cooing present
	Personal and social	Recognizes mother, social smile present
4	Developmental reflexes:	
	Spinal reflex	Presence of sucking, rooting, plantar, grasp, and Moro’s reflex
	Brainstem reflex	Absent
	Midbrain reactions	Absent
5	Motor examination:	
	Muscle tone	Hypotonia
	Sensory	Intact
	Reflexes	Exaggerated deep tendon reflexes bilaterally
	Tightness	Present of hamstring, tendon Achilles, and hip adductors

The motor tone assessments for both the right and left sides show consistent results. Shoulder flexors, shoulder abductors, elbow flexors, and wrist flexors all scored 1+ bilaterally. Similarly, hip flexors, hip abductors, knee flexors, and ankle plantar flexors also scored 1+ on both sides, as mentioned in Table 2.

**Table 2:** Muscle tone examination according to the TGS scale 1+: decreased response (hypotonia); TGS: tone grading scale

Muscle bulk	Right	Left
Shoulder flexor	1+	1+
Shoulder abductor	1+	1+
Elbow flexors	1+	1+
Wrist flexors	1+	1+
Hip flexors	1+	1+
Hip abductors	1+	1+
Knee flexors	1+	1+
Ankle plantar flexors	1+	1+

The deep tendon reflex (DTR) assessments indicate uniformly increased reflexes on both sides. The biceps jerk, triceps jerk, supinator jerk, knee jerk, and ankle jerk all scored +++ bilaterally. In addition, the plantar reflex is extensor on both the right and left sides, as mentioned in Table 3. 

**Table 3:** Reflex examination DTR: deep tendon reflexes; +++ : hyperreflexia

DTR	Biceps jerk	Triceps jerk	Supinator jerk	Knee jerk	Ankle jerk	Plantar reflex
Right	+++	+++	+++	+++	+++	Extensor
Left	+++	+++	+++	+++	+++	Extensor

Diagnostic assessment

**Figure 1:**
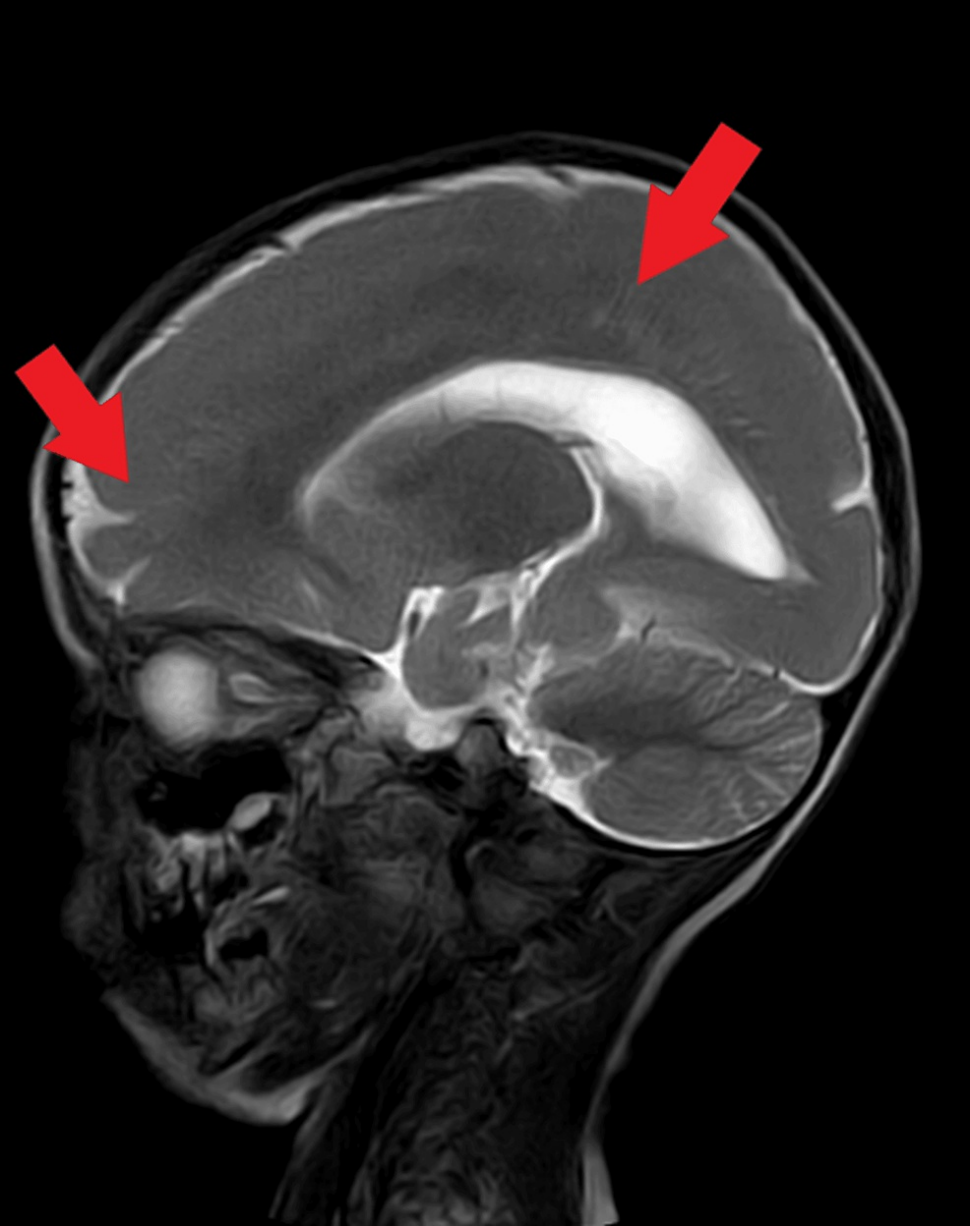
Magnetic resonance image demonstrating smoothing of sulci and gyri spaces in the brain, typically suggestive of type 1 lissencephaly

**Figure 2:**
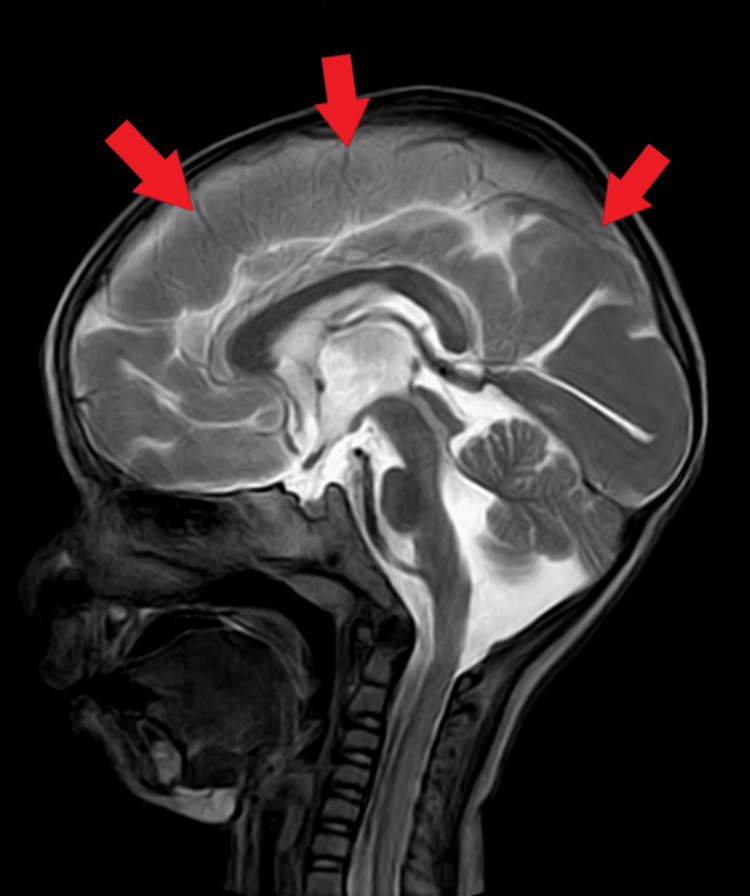
Magnetic resonance image demonstrating mild thickening of the cortex suggestive of type 1 lissencephaly

Electroencephalogram (EEG) report revealed the presence of intermittent repetitive sharp and high amplitude waves, which are in a generalized manner reflecting ongoing ictogenic discharges. The impression presents an abnormal EEG with generalized epileptogenesis. Brainstem evoked revoked audiometry test revealed, the formation of Vth wave formation at 35 decibels in both ears. MRI revealed mild thickening of the cortex and bahraigh diffusely involving the bilateral cerebral hemisphere suggestive of type 1 lissencephaly (Figures 1, 2).

Physiotherapy rehabilitation

The physiotherapy rehabilitation was commenced for 12 weeks, five days a week. The rehabilitation included numerous evidence-based approaches like neurodevelopmental technique, hippotherapy, and Rood's approach. At the end of the rehabilitation, positive results were obtained. Parent counseling was an integral part of the rehabilitation. The rehabilitation protocol is presented in Table 4. Neurodevelopmental techniques and hippotherapy were used for rehabilitation [13,14].

**Table 4:** Physiotherapy rehabilitation for the patient

Problem list	Physiotherapy goals	Therapeutic interventions
Lack of knowledge regarding the patient’s medical condition.	To educate patients regarding the understanding of the disease, and disease management	Importance of physiotherapy rehabilitation for the child. Counselling about the medical and therapeutic resources. Psychological counseling regarding emotional resilience, preparedness, and connecting with support groups. Counselling about the future planning for the parents.
Hypotonia of bilateral upper and lower limb muscles	To normalize the tone of the hypotonic muscles	Rood's facilitatory approach like tapping on muscle belly, tactile stimulation, quick stretching, mild stroking on the skin.
Partial head control of the patient	To initiate a symmetrical and upright neck-holding	Neurodevelopmental techniques to facilitate neck holding by stimulating neck extensors followed by counter-poising movements of the neck sideways.
Lack of postural stability	To improve postural stability	Vestibular ball exercises to initiate righting and equilibrium reactions.
Unable to sit without support	To initiate supported sitting followed by independent sitting	Neurodevelopmental techniques of trunk facilitation like transition from suoine to sitting, quadripod rocking techniques, reaching activities. Vestibular ball exercises to facilitate trunk control Hippo-therapy exercises. Perturbation exercises in sitting were commenced.
Difficulty in swallowing	To aid in swallowing	Oral sensorimotor stimulation and tactile stimulant to stimulate the gag reflex

## Discussion

The case study provides insights into the crucial role of physiotherapy rehabilitation in genetic disorders. Furthermore, it aims to increase knowledge about the pathophysiology of several genetic disorders and syndromes that physical therapists often treat. The goal is to provide an overview of the different facets of genetic disorders and how physical therapists address them. A major role for physical therapists can be played in the complex management of genetic illness. Patients with lissencephaly are prone to drug-resistant seizures. Even after the use of numerous anti-epileptic drugs, the majority of lissencephaly patients frequently do not show seizure control. According to Sandoval et al., newborns display uncontrollable epilepsy that begins weeks to months after birth [15]. Frequent epileptiform discharges have the potential to gradually exacerbate problems related to sensorimotor development and extensive cognitive function. In our case, no genetic methods were used; instead, the diagnosis was established based on radiological findings and clinical observations. Our case suggests type 1 lissencephaly based on the results. The syndrome has a very poor clinical prognosis with a life expectancy of about 10 years. Food aspiration, respiratory disorders, and severe seizures are the typical causes of death. Physiotherapy and other supportive treatments work to improve the quality of life and relieve symptoms associated with the disease.

According to Zanon et al., a therapist engages in dynamic interaction with a kid throughout the evaluation and treatment sessions. It is believed that through therapeutic handling and interaction, the child would eventually engage in meaningful activities and experience an improvement in their quality of life. This will also stimulate appropriate sensorimotor processing, task performance, and skill development [16]. The patient's internal reference systems are re-educated through afferent input, giving them more movement options and improved movement efficiency. It is a client-centered, hands‐on, problem-solving approach. It is used in the management and treatment of children who have disorders of function, movement, or postural control because of damage in their central nervous system. However, lissencephaly appears with specific phenotypic traits if the reelin pathway is compromised [17], including anterior predominance lissencephaly, severe cerebellar hypoplasia, and hippocampal abnormalities. Some of the most important steps in offering patients and their families the best counseling are determining the risk of recurrence and providing prognostic information to the multidisciplinary team that follows the patient in identifying the causative gene mutation of a rare disease [18]. Lissencephaly can be identified through ultrasonography at the first-second trimester scan [19]. Physical therapists assist in the development of children's movement competence and confidence by employing a physical literacy model that takes into account motor skills, motivation, and social and cognitive elements [20]. It is advised to use prenatal ultrasound for an early diagnosis to evaluate the situation and consider treatment options. The clinical outcome is often correlated with lisencephaly- subcortical band heterotopia severity grade, wherein intermediate pachygyria is less severe than severe agyria, and both are more severe than subcortical band heterotopia [21]. According to Lucas et al., task-oriented therapies with a home exercise program and a compliance log as reinforcement are the most effective for motor learning and skill acquisition [22].

## Conclusions

From this study, we deem the importance of physiotherapy rehabilitation in managing symptoms of genetic disorders. Physical rehabilitation plays a very pivotal role in improving the quality of life of patients. Our patient showed improvement in gaining delayed milestones and gained improvement in swallowing quality. Neurodevelopmental techniques, oral sensorimotor stimulation, and tactile stimulants worked to give positive outcomes. Our patient showed improvement in all the outcome measures used.
